# Special Issue: “Vaccination and Global Health”

**DOI:** 10.3390/vaccines12111223

**Published:** 2024-10-28

**Authors:** Shaodi Ma, Qian Bi, Li Liu, Roshan Thapa, Wenle Li, Baocheng Liu, Chuanhui Xu, Chenyu Sun

**Affiliations:** 1 Anhui Provincial Center for Disease Control and Prevention, Public Health Research Institute of Anhui Province, Hefei 230601, China; anyimsd@163.com; 2 Department of Radiation Oncology, State Key Laboratory of Complex Severe and Rare Diseases, Peking Union Medical College Hospital, Chinese Academy of Medical Sciences and Peking Union Medical College, Beijing 100050, China; pumc_iian@student.pumc.edu.cn; 3 The Second People’s Hospital of Hefei, Guangde Road, Hefei 230011, China; liulimbbs@163.com; 4 Hefei Hospital Affiliated to Anhui Medical University, Guangde Road, Hefei 230011, China; 5 General Internal Medicine, Medical College of Wisconsin, Milwaukee, WI 53226, USA; rosthapa@mcw.edu; 6 State Key Laboratory of Molecular Vaccinology and Molecular Diagnostics, Center for Molecular Imaging and Translational Medicine, School of Public Health, Xiamen University, Xiamen 361005, China; drlee0910@163.com; 7 Shanghai Innovation Center of Traditional Chinese Medicine Health Service, Shanghai University of Traditional Chinese Medicine, Shanghai 201203, China; baochliu@shutcm.edu.cn; 8 Department of Rheumatology, Allergy and Immunology, Tan Tock Seng Hospital, Singapore 308433, Singapore; xuchuanhui2008@gmail.com; 9 Lee Kong Chian School of Medicine, Nanyang Technological University, Singapore 545000, Singapore; 10 Department of General Surgery, The Second Affiliated Hospital of Anhui Medical University, Furong Road 678, Hefei 230601, China; 11 International Medical Department, The Second Affiliated Hospital of Anhui Medical University, Furong Road 678, Hefei 230601, China

## 1. Introduction

This Special Issue, titled ‘Vaccination and Global Health,’ compiles 11 broad-ranging papers, each exploring critical facets of vaccination, public health, and global healthcare systems [1,2,3,4,5,6,7,8,9,10,11]. These contributions add to the expanding body of research aimed at tackling the numerous challenges related to vaccination on a global scale. This Special Issue highlights the timeliness and significance of these contributions to the field. In this editorial, we provide a concise overview of the key findings from each paper, address the knowledge gaps filled by this Special Issue, and propose future research directions in this continuously evolving field.

## 2. Overview of Published Papers

The articles featured in this Special Issue cover a broad spectrum of topics, ranging from the development of novel vaccine technologies to the evaluation of vaccine efficacy across diverse populations. Several papers focus on the public health implications of vaccination programs, offering valuable insights into vaccine coverage, hesitancy, and the efficacy of global immunization efforts [3,4,5]. In one of the studies, Contoli et al. explores the willingness of foreign residents in Italy to be vaccinated, revealing the challenges faced by immigrant populations in terms of vaccine uptake. Their findings suggest the need for targeted communication strategies to increase vaccination rates in different communities [2]. Other articles examine the barriers to vaccine access in resource-limited settings and propose strategies to enhance global vaccine distribution [1,6]. Moreover, researchers also provide evidence for delivering vaccines as part of a humanitarian response by conducting a systematic review, which has contributed to the mapping of the normative landscape of vaccination guidance for vaccine-preventable diseases in crisis-affected settings [10].

A notable contribution is a study evaluating the implementation of COVID-19 vaccination programs in low- and middle-income countries [8]. The authors provide compelling evidence demonstrating the success of localized strategies tailored to specific regions. Another significant paper explores vaccine hesitancy by examining the socio-cultural factors that shaped public attitudes toward vaccination during the COVID-19 pandemic [4]. In addition, Kunieda et al. investigated the factors influencing measles vaccination rates in Niger [6], and recommended an increased focus on equity-based individual factors, including personal motivation, prompts and ability to access vaccination services. These studies provide valuable information and in-depth insights for enhancing vaccine acceptance globally, particularly through targeted educational campaigns and community engagement strategies [4,6].

## 3. Addressing Knowledge Gaps

This Special Issue addresses several pivotal knowledge gaps within the landscape of vaccination and global health. For example, several papers emphasize the challenges associated with ensuring equitable vaccine access, particularly among marginalized populations [1,8,9]. These findings are both timely and crucial, considering the persistent need to expand vaccine coverage in underserved regions. Additionally, research on the long-term efficacy of vaccines and their effectiveness against emerging disease variants offers valuable insights that can inform future vaccination strategies [11].

These articles provide evidence-based solutions that can be adopted by policymakers and public health officials to improve global vaccine distribution and uptake [1,2,3,4,5,6,7,8,9].

## 4. Future Research Directions

Looking ahead, several key areas of research merit further exploration. First, there is an urgent need to investigate the long-term efficacy of existing vaccines, particularly in the context of emerging variants of concern of COVID-19 [12,13,14,15,16,17,18,19,20,21,22,23,24,25,26,27]. Future research should also focus on the development of next-generation vaccines that are adaptable to a wider range of infectious diseases and capable of offering more durable protection.

Another critical area for future investigation is the optimization of vaccination strategies in regions where vaccine access continues to be a challenge [28,29,30,31,32,33,34,35,36,37,38,39,40,41,42,43,44,45,46,47,48,49,50,51,52,53,54,55,56,57,58,59,60,61,62,63,64,65,66]. This includes addressing not only logistical barriers but also cultural and socio-economic factors that influence vaccine acceptance. In addition, the role of digital technologies in supporting vaccination campaigns, from tracking vaccine distribution to combating misinformation, represents a promising area that could significantly enhance global vaccination efforts [67,68,69,70,71,72,73,74,75].

## 5. Conclusions

The articles in this Special Issue represent valuable contributions to the fields of vaccination and global health, addressing urgent challenges and proposing innovative solutions. The insights obtained from these studies will undoubtedly shape future public health strategies and research. We extend our sincere gratitude to the authors for their outstanding contributions and to the reviewers for their diligent efforts in upholding the high standards of this journal. We hope this Special Issue continues to inspire further research and action in the vital field of global vaccination.

## References

[1] Cheng L., Chan W.K., Zhu L., Chao M.H., Wang Y. (2024). Confronting Inequalities and Bridging the Divide: A Retrospective Study Assessment of Country-Level COVID-19 Vaccine Equality with a Cox Regression Model. Vaccines.

[2] Contoli B., Tosti M.E., Asta F., Minardi V., Marchetti G., Casigliani V., Scarso S., Declich S., Masocco M. (2024). Exploring COVID-19 Vaccination Willingness in Italy: A Focus on Resident Foreigners and Italians Using Data from PASSI and PASSI d’Argento Surveillance Systems. Vaccines.

[3] Ahmad M., Sattar A., Aroosa S., Majeed A., Rasheed M.A., Ahmad W., Iqbal A., Omer M.O., Beg B.M., Mushtaq R.M.Z. (2023). Attitude and Acceptance towards COVID-19 Booster Doses among Literacy Advantaged Population in Pakistan: A Cross-Sectional Study. Vaccines.

[4] Daghriri T., Proctor M., Matthews S., Bashiri A.H. (2023). Modeling Behavior and Vaccine Hesitancy Using Twitter-Derived US Population Sentiment during the COVID-19 Pandemic to Predict Daily Vaccination Inoculations. Vaccines.

[5] Osaghae I., Darkoh C., Chido-Amajuoyi O.G., Chan W., Padgett Wermuth P., Pande M., Cunningham S.A., Shete S. (2023). Healthcare Provider’s Perceived Self-Efficacy in HPV Vaccination Hesitancy Counseling and HPV Vaccination Acceptance. Vaccines.

[6] Kunieda M.K., Manzo M.L., Subramanian S.V., Jimba M. (2022). Individual- and Neighborhood-Level Factors of Measles Vaccination Coverage in Niamey, Niger: A Multilevel Analysis. Vaccines.

[7] Yap R.X.L., Lai Y.W., Wei C., Ng J.J.W., Xu D., Feng S., Mu R., Thong B.Y., Xu C. (2024). Impact of Immunomodulatory Therapy on COVID-19 Vaccine Response in Patients with Autoimmune Inflammatory Rheumatic Diseases. Vaccines.

[8] Belt R.V., Abdullah S., Mounier-Jack S., Sodha  S.V., Danielson N., Dadari I., Olayinka F., Ray A., Crocker-Buque T. (2023). Improving Equity in Urban Immunization in Low- and Middle-Income Countries: A Qualitative Document Review. Vaccines.

[9] Ayyalasomayajula S., Dhawan A., Karattuthodi M.S., Thorakkattil S.A., Abdulsalim S., Elnaem M.H., Sridhar S., Unnikrishnan M.K. (2023). A Systematic Review on Sociodemographic, Financial and Psychological Factors Associated with COVID-19 Vaccine Booster Hesitancy among Adult Population. Vaccines.

[10] Allison L.E., Alhaffar M., Checchi F., Abdelmagid  N.,  Nor B., Sabahelzain M.M., Light P.M., Singh N.S. (2023). A Systematic Review of Vaccination Guidance for Humanitarian Responses. Vaccines.

[11] Liang H.Y., Wu Y., Yau V., Yin H.X., Lowe S., Bentley R., Ahmed M.A., Zhao W., Sun  C. (2022). SARS-CoV-2 Variants, Current Vaccines and Therapeutic Implications for COVID-19. Vaccines.

[12] Lin D.Y., Zeng D., Gilbert P.B. (2021). Evaluating the Long-term Efficacy of Coronavirus Disease 2019 (COVID-19) Vaccines. Clin. Infect. Dis..

[13] Tandler C., Heitmann J.S., Michel T.M., Marconato M., Jaeger  S.U., Tegeler C.M., Denk M., Richter M., Oezbek M.T., Maringer Y. (2024). Long-term efficacy of the peptide-based COVID-19 T cell activator CoVac-1 in healthy adults. Int. J. Infect. Dis..

[14] Miazga W., Wnuk K., Tatara T., Świtalski J., Matera A., Religioni U., Gujski M. (2023). The long-term efficacy of tick-borne encephalitis vaccines available in Europe—A systematic review. BMC Infect Dis..

[15] Chen X., Wang W., Chen X., Wu Q., Sun R., Ge S., Zheng N., Lu W., Yang J., Rodewald L. (2022). Prediction of long-term kinetics of vaccine-elicited neutralizing antibody and time-varying vaccine-specific efficacy against the SARS-CoV-2 Delta variant by clinical endpoint. BMC Med..

[16] Nasreen S., Chung H., He S., Brown K.A., Gubbay J.B., Buchan S.A., Fell D.B., Austin P.C., Schwartz K.L., Sundaram M.E. (2022). Effectiveness of COVID-19 vaccines against symptomatic SARS-CoV-2 infection and severe outcomes with variants of concern in Ontario. Nat. Microbiol..

[17] Pouwels K.B., Pritchard E., Matthews P.C., Stoesser N., Eyre D.W., Vihta K.D., House T., Hay J., Bell  J.I., Newton J.N. (2021). Effect of Delta variant on viral burden and vaccine effectiveness against new SARS-CoV-2 infections in the UK. Nat. Med..

[18] Sheikh A., McMenamin J., Taylor B., Robertson C., Public Health Scotland and the EAVE II Collaborators (2021). SARS-CoV-2 Delta VOC in Scotland: Demographics, risk of hospital admission, and vaccine effectiveness. Lancet.

[19] Reis B.Y., Barda N., Leshchinsky M., Kepten E., Hernán M.A., Lipsitch M., Dagan N., Balicer R.D. (2021). Effectiveness of BNT162b2 Vaccine against Delta Variant in Adolescents. N. Engl. J. Med..

[20] Li X.N., Huang Y., Wang W., Jing Q.L., Zhang C.H., Qin P.Z., Guan W.J., Gan L., Li Y.L., Liu W.H. (2021). Effectiveness of inactivated SARS-CoV-2 vaccines against the Delta variant infection in Guangzhou: A test-negative case-control real-world study. Emerg. Microbes. Infect..

[21] McKeigue P.M., McAllister D.A., Hutchinson S.J., Robertson C., Stockton D., Colhoun H.M. (2022). Vaccine efficacy against severe COVID-19 in relation to delta variant (B.1.617.2) and time since second dose in patients in Scotland (REACT-SCOT): A case-control study. Lancet Respir. Med..

[22] Ella R., Reddy S., Blackwelder W., Potdar V., Yadav P., Sarangi V., Aileni V.K., Kanungo S., Rai S., Reddy P. (2021). Efficacy, safety, and lot-to-lot immunogenicity of an inactivated SARS-CoV-2 vaccine (BBV152): Interim results of a randomised, double-blind, controlled, phase 3 trial. Lancet.

[23] Barlow R.S., Larson L., Jian K. (2021). Effectiveness of COVID-19 vaccines against SARS-CoV-2 infection during a Delta variant epidemic surge in Multnomah County, Oregon, July 2021. medRxiv.

[24] Tenforde M.W., Self W.H., Naioti E.A., Ginde A.A., Douin D.J., Olson S.M., Talbot H.K., Casey J.D., Mohr N.M., Zepeski A. (2021). Sustained Effectiveness of Pfizer-BioNTech and Moderna Vaccines Against COVID-19 Associated Hospitalizations Among Adults—United States, March-July 2021. MMWR Morb. Mortal. Wkly. Rep..

[25] Chia P.Y., Ong S.W.X., Chiew C.J., Ang L.W., Chavatte J.M., Mak T.M., Cui L., Kalimuddin S., Chia W.N., Tan C.W. (2022). Virological and serological kinetics of SARS-CoV-2 Delta variant vaccine breakthrough infections: A multicentre cohort study. Clin. Microbiol. Infect..

[26] Keegan L., Truelove S.A., Lessler J. (2021). Progress of the Delta variant and erosion of vaccine effectiveness, a warning from Utah. medRxiv.

[27] Tartof S.Y., Slezak J.M., Fischer H., Hong V., Ackerson B.K., Ranasinghe O.N., Frankland T.B., Ogun O.A., Zamparo J.M., Gray S. (2021). Effectiveness of mRNA BNT162b2 COVID-19 vaccine up to 6 months in a large integrated health system in the USA: A retrospective cohort study. Lancet.

[28] Pennisi F., Genovese C., Gianfredi V. (2024). Lessons from the COVID-19 Pandemic: Promoting Vaccination and Public Health Resilience, a Narrative Review. Vaccines.

[29] Kyung S., Rahmati M., Kang J., Lee K., Lee H., Yon D.K. (2024). Global and Regional Burden of Vaccine-Associated Erythema Multiforme and their Related Vaccines, 1967-2023: An In-Depth Analysis of the World Health Organization Pharmacovigilance Database. Med. Princ. Pract..

[30] Miyazato Y., Terada M., Ujiie M., Lee K., Lee H., Yon D.K. (2023). A nationwide prospective cohort study on safety of the 17D-204 yellow fever vaccine during a vaccine shortage in Japan. J. Travel. Med..

[31] Wong N.X., Buttery J., McMinn A., Azhar Z., Crawford N.W. (2020). Safety of the Polish BCG-10 Vaccine During a Period of BCG Vaccine Shortage: An Australian Experience. Pediatr. Infect. Dis. J..

[32] Groom A.V., Cheek J.E., Bryan R.T. (2006). Effect of a national vaccine shortage on vaccine coverage for American Indian/Alaska Native children. Am. J. Public Health.

[33] Flavia A., Fred B., Eleanor T. (2023). Gaps in vaccine management practices during vaccination outreach sessions in rural settings in southwestern Uganda. BMC Infect. Dis..

[34] Feinmann J. (2024). Vaccine shortages worsen the deadliest cholera outbreaks in years. BMJ.

[35] Miranda-García M.A., Hoffelner M., Stoll H., Ruhaltinger D., Cichutek K., Siedler A., Bekeredjian-Ding I. (2022). A 5-year look-back at the notification and management of vaccine supply shortages in Germany. Euro. Surveill..

[36] Kempe A., Daley M.F., Stokley S., Crane L.A., Beaty B.L., Barrow J., Babbel C., Dickinson L.M., Steiner J.F., Berman S. (2007). Impact of a severe influenza vaccine shortage on primary care practice. Am. J. Prev. Med..

[37] Glatman-Freedman A., Cohen M.L., Nichols K.A., Porges R.F., Saludes I.R., Steffens K., Rodwin V.G., Britt D.W. (2010). Factors affecting the introduction of new vaccines to poor nations: A comparative study of the Haemophilus influenzae type B and hepatitis B vaccines. PLoS ONE.

[38] Gaitán-Rossi P., Mendez-Rosenzweig M., García-Alberto E., Vilar-Compte M. (2022). Barriers to COVID-19 vaccination among older adults in Mexico City. Int. J. Equity. Health.

[39] Jammeh A., Muhoozi M., Kulane A., Kajungu D. (2023). Comparing full immunisation status of children (0-23 months) between slums of Kampala City and the rural setting of Iganga District in Uganda: A cross-sectional study. BMC Health Serv. Res..

[40] Vasudevan L., Baumgartner J.N., Moses S., Ngadaya E., Mfinanga S.G., Ostermann J. (2020). Parental concerns and uptake of childhood vaccines in rural Tanzania—A mixed methods study. BMC Public Health.

[41] Babalola S. (2011). Maternal reasons for non-immunisation and partial immunisation in northern Nigeria. J. Paediatr. Child Health.

[42] Abdulraheem I.S., Onajole A.T., Jimoh A.A.G., Oladipo A.R. (2011). Reasons for incomplete vaccination and factors for missed opportunities among rural Nigerian children. J. Public Health Epidemiol..

[43] Abebe A.M., Mengistu T., Mekuria A.D. (2019). Measles case, immunization coverage and its determinant factors among 12-23 month children, in Bassona Worena Woreda, Amhara Region, Ethiopia, 2018. BMC Res. Notes..

[44] Adamu A.A., Uthman O.A., Gadanya M.A., Cooper S., Wiysonge C.S. (2019). Using the theoretical domains framework to explore reasons for missed opportunities for vaccination among children in Kano, Nigeria: A qualitative study in the pre-implementation phase of a collaborative quality improvement project. Expert Rev. Vaccines..

[45] Atwiine B., Rukundo A., Elias B., MacDonald N.E. (2016). Reasons for non-timely completion of the routine infant immunization schedule by children in rural South West Uganda. Can. J. Public Health.

[46] Babirye J.N., Rutebemberwa E., Kiguli J., Wamani H., Nuwaha F., Engebretsen I.M. (2011). More support for mothers: A qualitative study on factors affecting immunisation behaviour in Kampala, Uganda. BMC Public Health.

[47] Babirye J.N., Engebretsen I.M., Rutebemberwa E., Kiguli J., Nuwaha F. (2014). Urban settings do not ensure access to services: Findings from the immunisation programme in Kampala Uganda. BMC Health Serv. Res..

[48] Cockcroft A., Usman M.U., Nyamucherera O.F., Emori  H., Duke B., Umar N.A., Andersson N. (2014). Why children are not vaccinated against measles: A cross-sectional study in two Nigerian States. Arch Public Health.

[49] Holte J.H., Mæstad O., Jani J.V. (2012). The decision to vaccinate a child: An economic perspective from southern Malawi. Soc. Sci. Med..

[50] Itimi K., Dienye P.O., Ordinioha B. (2012). Community participation and childhood immunization coverage: A comparative study of rural and urban communities of Bayelsa State, south-south Nigeria. Niger. Med. J..

[51] Kagone M., Ye M., Nebie E., Sié A., Müller O., Beiersmann C. (2018). Community perception regarding childhood vaccinations and its implications for effectiveness: A qualitative study in rural Burkina Faso. BMC Public Health.

[52] Kwedi Nolna S., Bonono C.R., Nsangou Moncher M., Bindé T., Nolna D., Ongolo Zogo P. (2018). Factors influencing the performance of routine immunization in urban areas: A comparative case study of two cities in Cameroon: Douala and Yaoundé. Vaccine.

[53] LaMontagne D.S., Barge S., Le N.T., Mugisha E., Penny M.E., Gandhi S., Janmohamed A., Kumakech E., Mosqueira N.R., Nguyen N.Q. (2011). Human papillomavirus vaccine delivery strategies that achieved high coverage in low- and middle-income countries. Bull World Health Organ..

[54] Mbabazi W., Lako A.K., Ngemera D., Laku R., Yehia M., Nshakira N. (2013). Maiden immunization coverage survey in the republic of South Sudan: A cross-sectional study providing baselines for future performance measurement. Pan. Afr. Med. J..

[55] Mekonnen A.G., Bayleyegn A.D., Ayele E.T. (2019). Immunization coverage of 12-23 months old children and its associated factors in Minjar-Shenkora district, Ethiopia: A community-based study. BMC Pediatr..

[56] Meyer S.A., Kambou J.L., Cohn A., Goodson J.L., Flannery B., Medah I., Messonnier N., Novak R., Diomande F., Djingarey M.H. (2015). Serogroup A meningococcal conjugate (PsA-TT) vaccine coverage and measles vaccine coverage in Burkina Faso–implications for introduction of PsA-TT into the Expanded Programme on Immunization. Vaccine.

[57] Michael C.A., Ashenafi S., Ogbuanu I.U., Ohuabunwo C., Sule A., Corkum M., Mackay S., Storms A.D., Achari P., Biya O. (2014). An evaluation of community perspectives and contributing factors to missed children during an oral polio vaccination campaign–Katsina State, Nigeria. J. Infect. Dis..

[58] Michael C.A., Ogbuanu I.U., Storms A.D., Ohuabunwo C.J., Corkum M., Ashenafi S., Achari P., Biya O., Nguku P., Mahoney F. (2014). An assessment of the reasons for oral poliovirus vaccine refusals in Northern Nigeria. J. Infect. Dis..

[59] Msyamboza K.P., Mwagomba B.M., Valle M., Chiumia H., Phiri T. (2017). Implementation of a human papillomavirus vaccination demonstration project in Malawi: Successes and challenges. BMC Public Health.

[60] Murele B., Vaz R., Gasasira A., Mkanda P., Erbeto T., Okeibunor J. (2014). Vaccine perception among acceptors and non-acceptors in Sokoto State, Nigeria. Vaccine.

[61] Nabirye J., Okwi L.A., Nuwematsiko R., Kiwanuka G., Muneza F., Kamya C., Babirye J.N. (2020). Health system factors influencing uptake of Human Papilloma Virus (HPV) vaccine among adolescent girls 9-15 years in Mbale District, Uganda. BMC Public Health.

[62] Nguefack Dongmo F., Tassadong C., Dongmo R., Tatah S., Fodoung W.D.S., Chiabi A., Kago I., Kobela M. (2016). Factors influencing routine vaccination of children of mothers live-stock retailers in the markets of Yaoundé. World J. Vaccin..

[63] Njeru I., Ajack Y., Muitherero C., Onyango D., Musyoka J., Onuekusi I., Kioko J., Muraguri N., Davis R. (2016). Did the call for boycott by the Catholic bishops affect the polio vaccination coverage in Kenya in 2015? a cross-sectional study. Pan. Afr. Med. J..

[64] Nsubuga F., Kabwama S.N., Ampeire I., Luzze H.,  Gerald P., Bulage L., Toliva O.B. (2019). Comparing static and outreach immunization strategies and associated factors in Uganda, Nov-Dec 2016. Pan. Afr. Med. J..

[65] Okenwa U.J., Dairo M.D., Uba B., Ajumobi O. (2019). Maternal reasons for non-receipt of valid Hepatitis B birth dose among mother-infant pairs attending routine immunization clinics, South-east, Nigeria. Vaccine.

[66] Oladokun R.E., Adedokun B.O., Lawoyin T.O. (2010). Children not receiving adequate immunization in Ibadan, Nigeria: What reasons and beliefs do their mothers have?. Niger. J. Clin. Pract..

[67] Atkinson K., Ntacyabukura B., Hawken S., El-Khatib Z., Laflamme L., Wilson K. (2024). Parent and family characteristics associated with reported pediatric influenza vaccination in a sample of Canadian digital vaccination platform users. An exploratory, cross-sectional study in the 2018-2019 influenza season. Hum. Vaccin. Immunother..

[68] Kim K., Parekh De Campos A., Choi S. (2022). A Vax4HPV Mobile Application for Parents of Human Papillomavirus Vaccine-Eligible Children: Iterative Formative Assessments. Comput. Inform. Nurs..

[69] Shegog R., Savas L.S., Healy C.M., Frost E.L., Coan S.P., Gabay E.K., Preston S.M., Spinner S.W., Wilbur M., Becker E. (2022). AVPCancerFree: Impact of a digital behavior change intervention on parental HPV vaccine -related perceptions and behaviors. Hum. Vaccin. Immunother..

[70] Ortiz R.R., Shafer A., Cates J., Coyne-Beasley T. (2018). Development and Evaluation of a Social Media Health Intervention to Improve Adolescents’ Knowledge About and Vaccination Against the Human Papillomavirus. Glob. Pediatr. Health..

[71] Buller D.B., Pagoto S., Henry K., Berteletti J., Walkosz B.J., Bibeau J., Baker K., Hillhouse J., Arroyo K.M. (2021). Human Papillomavirus Vaccination and Social Media: Results in a Trial With Mothers of Daughters Aged 14-17. Front. Digit. Health.

[72] Chodick G., Teper G.R., Levi S., Kopel H., Kleinbort A., Khen E., Schejter E., Shalev V., Stein M., Lewis N. (2021). The impact of a Facebook campaign among mothers on HPV vaccine uptake among their daughters: A randomized field study. Gynecol. Oncol..

[73] Occa A., Stahl H.M., Julien-Bell S. (2022). Helping Children to Participate in Human Papillomavirus-Related Discussions: Mixed Methods Study of Multimedia Messages. JMIR Form Res..

[74] Chen A.C., Kim W.S., Todd M., Larkey L. (2022). A Digital Storytelling Intervention for Vietnamese American Mothers to Promote Their Children’s HPV Vaccination. Cancer Prev. Res..

[75] Woodall W.G., Zimet G., Kong A., Buller D., Reither J., Chilton L., Myers V., Starling R. (2021). Vacteens.org: A Mobile Web app to Improve HPV Vaccine Uptake. Front. Digit. Health.

